# Pacific-Ciguatoxin-2 and Brevetoxin-1 Induce the Sensitization of Sensory Receptors Mediating Pain and Pruritus in Sensory Neurons

**DOI:** 10.3390/md19070387

**Published:** 2021-07-06

**Authors:** Ophélie Pierre, Maxime Fouchard, Nelig Le Goux, Paul Buscaglia, Raphaël Leschiera, Richard J. Lewis, Olivier Mignen, Joachim W. Fluhr, Laurent Misery, Raphaële Le Garrec

**Affiliations:** 1 Laboratoire Interactions Epithéliums-Neurones (LIEN), University of Brest, EA4685, F-29200 Brest, France; Maxime.FOUCHARD@chu-rennes.fr (M.F.); raphael.leschiera@univ-brest.fr (R.L.); Joachim.Fluhr@charite.de (J.W.F.); Laurent.misery@chu-brest.fr (L.M.); rlegarrec@univ-brest.fr (R.L.G.); 2 Department of Dermatology, University Hospital of Brest, F-29200 Brest, France; 3 Lymphocytes B et Autoimmunité, Faculty of Medicine and Health Sciences, University of Brest, Inserm, UMR1227, F-29200 Brest, France; Nelig.LeGoux@univ-brest.fr (N.L.G.); paul-buscaglia@uiowa.edu (P.B.); olivier.mignen@univ-brest.fr (O.M.); 4 Department of Molecular Physiology and Biophysics, Fraternal Order of Eagle Diabetes Research Center, Iowa Neuroscience Institute, Pappajohn Biomedical Institute, University of Iowa, Iowa City, IA 52242, USA; 5 Institute for Molecular Bioscience, The University of Queensland, St. Lucia, QLD 4072, Australia; r.lewis@uq.edu.au; 6 Department of Dermatology and Allergology, Universitaetsmedizin Charité Berlin, D-10117 Berlin, Germany

**Keywords:** ciguatoxin, ciguatera fish poisoning, brevetoxin, neurotoxic shellfish poisoning, sensory disorders, pruritus, pain, sensitization, receptors, ion channels

## Abstract

Ciguatera fish poisoning (CFP) and neurotoxic shellfish poisoning syndromes are induced by the consumption of seafood contaminated by ciguatoxins and brevetoxins. Both toxins cause sensory symptoms such as paresthesia, cold dysesthesia and painful disorders. An intense pruritus, which may become chronic, occurs also in CFP. No curative treatment is available and the pathophysiology is not fully elucidated. Here we conducted single-cell calcium video-imaging experiments in sensory neurons from newborn rats to study in vitro the ability of Pacific-ciguatoxin-2 (P-CTX-2) and brevetoxin-1 (PbTx-1) to sensitize receptors and ion channels, (i.e., to increase the percentage of responding cells and/or the response amplitude to their pharmacological agonists). In addition, we studied the neurotrophin release in sensory neurons co-cultured with keratinocytes after exposure to P-CTX-2. Our results show that P-CTX-2 induced the sensitization of TRPA1, TRPV4, PAR2, MrgprC, MrgprA and TTX-r NaV channels in sensory neurons. P-CTX-2 increased the release of nerve growth factor and brain-derived neurotrophic factor in the co-culture supernatant, suggesting that those neurotrophins could contribute to the sensitization of the aforementioned receptors and channels. Our results suggest the potential role of sensitization of sensory receptors/ion channels in the induction or persistence of sensory disturbances in CFP syndrome.

## 1. Introduction

Ciguatoxins (CTX) are produced by dinoflagellates of the genera *Gambierdiscus* and *Fukuyoa* [1,2,3,4,5] and bioaccumulate in tropical and sub-tropical fishes [1,6]. Ciguatera fish poisoning (CFP) is a syndrome consecutive to an oral consumption of CTX contaminated fish [6,7]. Between 50,000 and 500,000 persons are estimated to be affected annually [6,8,9,10]. Clinically, CFP includes gastrointestinal symptoms (e.g., diarrhea, nausea, vomiting and abdominal pain), typical sensory disturbances (e.g., paresthesia, cold dysesthesia, pruritus, dental pain and myalgia) and in the most severe cases, cardiovascular signs [7,11,12,13,14,15,16,17,18]. The intense and disabling sensory disturbances of CFP [11,19], can persist several days, weeks or even years after the toxic meal ingestion. In some cases, sensory symptoms reoccur or are exacerbated after consumption of alcohol, certain foods or after physical exercise [6,20]. No curative treatment is available and the pathophysiology of the sensory disturbances is not fully understood.

CTX target mainly voltage gated sodium channels (NaV) [21], which are highly expressed in the dorsal root ganglion (DRG) neurons. Brevetoxins, responsible for neurotoxic shellfish poisoning (NSP) considered as a moderate CFP, are structural and functional analogs of CTX that also target the NaV channels. Toxin binding to NaV channels enhances a sodium influx which leads to membrane hyperexcitability [22,23,24,25] that can lead to sensory disturbance. Previously, we have shown that Pacific-ciguatoxin-2 (P-CTX-2) and brevetoxin-1 (PbTx-1) induce neuropeptide (substance P (SP) and calcitonin gene-related peptide (CGRP)) release from a co-culture of sensory neurons and differentiated keratinocytes [26,27,28]. Other studies demonstrated a neuropeptide release from murine skin treated with Pacific-ciguatoxin-1 (P-CTX-1) [12,29]. In skin, the release of neuropeptides drives cutaneous neurogenic inflammation characterized by flare, edema and pruritus [30], signs also observed in CFP cases [7] and P-CTX-1 intracutaneous injection in humans [12]. In the central nervous system, neuropeptides mediate pain and itch transmission [31,32,33] and contribute to both peripheral and central sensitization [34].

Pain and itch are two distinct sensations with their own dedicated sensory pathways but common mechanisms. Sensitization of sensory receptors or ion channels is one of the mechanisms resulting in chronic pain as well as chronic itch. It is defined as a reduction of activation threshold and/or an increase of the response amplitude to a stimulus. Namely, normally ineffective stimuli might be effective or a spontaneous activation can be induced. Sensitization of sensory receptors/ion channels is well established in inflammatory, neuropathic pain [35] and can occur both centrally and peripherally. A combination of both is frequently present in chronic pain and itch. Sensitizing mediators include proinflammatory cytokines, neuropeptides, bradykinins, chemokines, histamine and neurotrophins (nerve growth factor (NGF)), brain-derived neurotrophic factor (BDNF) and glial cell line-derived neurotrophic factor [36,37,38]. Those mediators bind to their receptors whose signaling induces the sensitization of sensory receptors and/or ion channels. Different molecular mechanisms are involved, including the activation of protein kinases and an increase in trafficking and/or gene expression [39,40,41]. We focused on the neurotrophins, including NGF and BDNF which are the most commonly studied growth factors in the sensitization of receptors [42,43], given the important role they play in the processing of nociceptive/pruriceptive information in the periphery and/or the central nervous system [44,45].

Previous work showed that a vast majority of murine sensory neurons responding to 1 nM of P-CTX-1 express transient receptor potential A1 (TRPA1) [46]. Cold allodynia after CTX intraplantar injection in mice has been shown to involve the sensitization to cold of tetrodotoxin-sensitive (TTX-s) A-fibres and TRPA1 in NaV1.8-expressing C-fibers [46]. Sensitization of channels/receptors expressed in sensory neurons contributes to the development and maintenance of pain and/or pruritus [35]. TTX-s and TTX-resistant (TTX-r) NaV channels, which are highly expressed in the peripheral nervous system [47], are the main targets of CTX and PbTx and can be sensitized [48]. Other channels including transient receptor potential vanilloid 1 (TRPV1) [49,50,51,52], transient receptor potential vanilloid 4 (TRPV4) [53] and TRPA1 [40,54] are involved in pruritus and several types of chronic pain [55,56]. Protease-activated receptor 2 (PAR2), mainly expressed by small diameter fibers [57], is involved in pruritus [58] and peripheral neuropathy induced by chemotherapy [59,60,61]. Previously, we have shown that PAR2 is activated by P-CTX-2 and PbTx-1 in sensory neurons [26,28]. PAR2 is known to sensitize transient receptor potential (TRP) channels [53,54,55,62]. Mas-related G-protein coupled receptors (Mrgpr), including MrgprA and MrgprC, expressed in unmyelinated fibers innervating epidermis, were recently identified as itch-sensing receptors [63]. PAR2 activation potentiated itch induced by their agonists in the mouse model, suggesting sensitization [64]. Thus, we were interested in studying the sensitization of TRP channels, PAR2 and Mrgprs by CTX and PbTx.

Our hypothesis was that the sensitization of sensory receptors/ion channels by CTX and PbTx contributes to the development and maintenance of CFP and NSP sensory symptoms. In this new work, we studied the ability of P-CTX-2 and PbTx-1 to sensitize receptors/ion channels including TRPA1, TRPV1, TRPV4, PAR2, MrgprC, MrgprA, and TTX-r NaV channels after a 20 h exposure. We used single-cell calcium video imaging in newborn rat sensory neurons by measuring the toxin ability to increase the percentage of responding cells and/or the response amplitude to the pharmacological agonist of each receptor/channel. In addition, we explored the ability of P-CTX-2 to increase NGF and BDNF release by quantifying neurotrophin levels in the supernatant of co-culture of sensory neurons and differentiated keratinocytes following a 24 h exposure to P-CTX-2. Our data provide new knowledge on the physiopathology of sensory symptoms induced by CTX and PbTx. These results could be useful for the development of specific treatments.

## 2. Results

We studied the ability of P-CTX-2 and PbTx-1 to sensitize several receptors and ion channels in DRG neurons using single-cell calcium video imaging. After pretreatment with PbTx-1, P-CTX-2 or vehicle controls for 20 h, we assessed the change in the calcium response (percentage of responding cells and/or the response amplitude) to the specific agonist of each sensory receptor (e.g., capsaicin for TRPV1) induced by the toxins compared with their vehicle controls.

### 2.1. Potentiation of the Calcium Response of Sensory Receptors/Channels to their Agonist after PbTx-1 or P-CTX-2 Pretreatment

**Figure 1:**
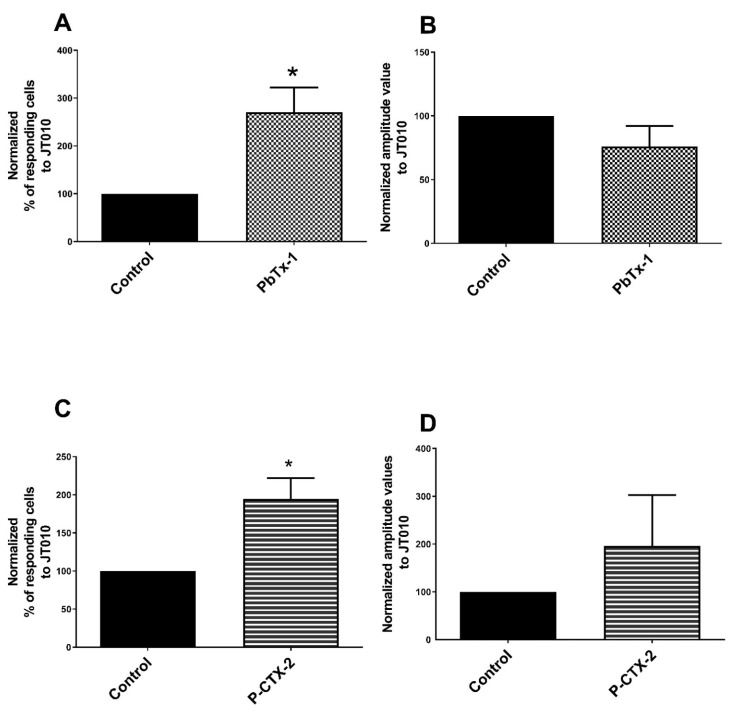
Sensitization of TRPA1 by PbTx-1 and P-CTX-2 in DRG neurons. Sensory neurons were pretreated with 10 nM of P-CTX-2, 1 µM of PbTx-1 or vehicles for 20 h. Specific agonist of TRPA1 (JT010) was used to assess the sensitization of this channel. Normalized percentages of responding cells (**A**,**C**) and the normalized associated amplitude values (**B**,**D**) of the calcium signal in response to the JT010 were recorded. Normalized data were obtained from 3 (with PbTx-1) and 5 (with P-CTX-2) independent experiments and data were expressed as mean ± SEM. * *p* < 0.05. DRG: dorsal root ganglion; PbTx-1: brevetoxin-1; P-CTX-2: Pacific-ciguatoxin-2; TRPA1: transient receptor potential ankyrin 1.

We assessed the ability of the toxins to sensitize TRPA1 using the agonist JT010 [65]. The percentage of cells responding to JT010 was significantly increased after pretreatment with PbTx-1 (271 ± 51% compared to control (100%)) (Figure 1A) and after pretreatment with P-CTX-2 (195 ± 27% compared to control (100%)) (Figure 1C). In contrast, PbTx-1 and P-CTX-2 did not significantly modify the amplitude values. A strong increase in the amplitude values were recorded after pretreatment with P-CTX-2 although this did not reach the significance level (196 ± 48% compared to control (100%)) (Figure 1B,D). These results suggest a sensitization of TRPA1 by P-CTX-2 and PbTx-1 in sensory neurons.

**Figure 2:**
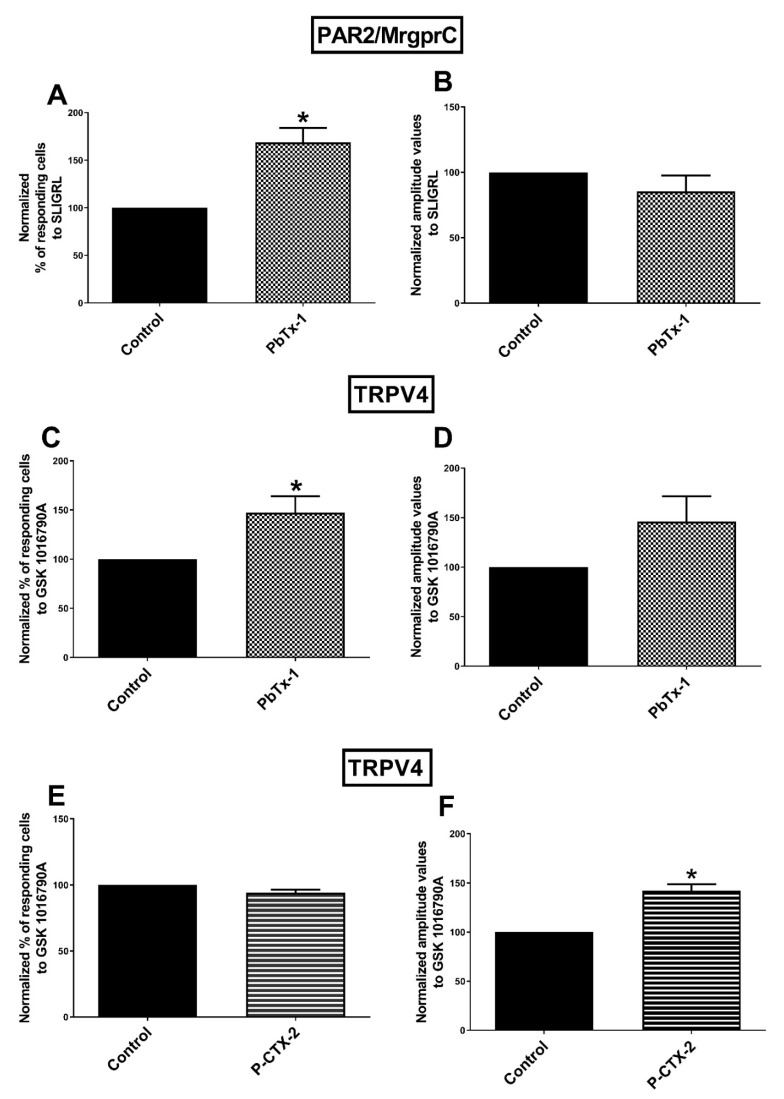
Sensitization of PAR2/MrgprC by PbTx-1 and TRPV4 by PbTx-1 and P-CTX-2 in DRG neurons. Sensory neurons were pretreated with 10 nM of P-CTX-2, 1 µM of PbTx-1 or vehicle for 20 h. Peptide activator of PAR2 and MrgprC (SLIGRL) and agonist of TRPV4 (GSK 1016790A) were used to assess sensitization of these channels/receptors. Normalized percentages of responding cells (**A**,**C**,**E**) and the associated amplitude values (**B**,**D**,**F**) of the calcium signal in response to the specific agonist were recorded. Normalized data were obtained from 5 (PAR2 and MrgprC), 6 (TRPV4 in PbTx-1 condition) and 3 (TRPV4 in P-CTX-2 condition) independent experiments. Data were expressed as mean ± SEM. * *p* < 0.05. DRG: dorsal root ganglion; MrgprC: Mas-related G-protein coupled receptors C; PAR2: protease-activated receptor 2; PbTx-1: brevetoxin-1; P-CTX-2: Pacific-ciguatoxin-2; TRPV4: transient receptor potential vanilloid 4.

SLIGRL is able to activate PAR2 [66] and MrgprC [67]. We assessed the ability of PbTx-1 and P-CTX-2 to sensitize PAR2/MrgprC and TRPV4 using the peptide activators SLIGRL and GSK-1016790A [68], respectively. The percentage of responding cells to the SLIGRL and GSK-1016790A were significantly enhanced after pretreatment with PbTx-1 (169 ± 15% and 147 ± 17% of control, respectively). However, no significant modification of the amplitude values was recorded (85 ± 12% and 146 ± 25% of controls, respectively) (Figure 2A−D). The percentage of responding cells to GSK-1016790A after P-CTX-2 pretreatment was not modified (94 ± 2.3% of the control) (Figure 2E), while the amplitude values recorded for TRPV4 were significantly increased compared to control (142 ± 6.3%) (Figure 2F). These results suggest that PAR2/MrgprC and TRPV4 are sensitized by PbTx-1 in sensory neurons. Similarly, P-CTX-2 sensitized TRPV4, while its effect on SLIGRL response (PAR2 and MrgprC) was not assessed.

**Figure 3:**
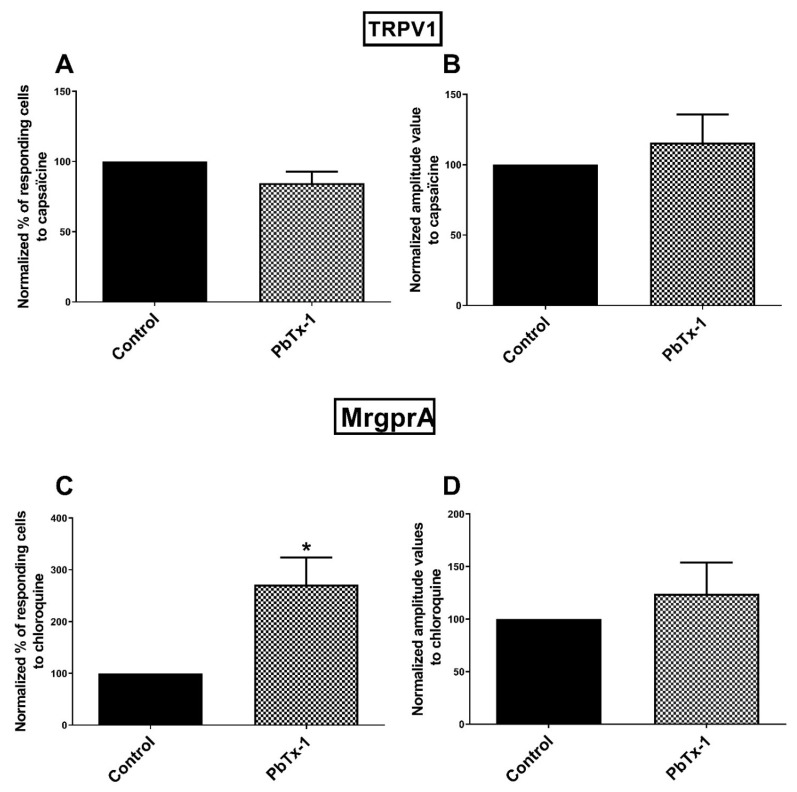
Sensitization of TRPV1 and MrgprA by PbTx-1 in DRG neurons. Sensory neurons were pretreated with 1 µM of PbTx-1 or its vehicle for 20 h. Specific agonists of TRPV1 (capsaicin) and MrgprA (chloroquine) were used to assess the sensitization of these channels/receptors. Normalized percentages of responding cells (**A**,**C**) and the associated amplitude values (**B**,**D**) of the calcium signal were recorded. Normalized data were obtained from 9 (TRPV1) and 5 (MrgprA) independent experiments. Statistical analysis has been performed using a t-test and data were expressed as mean ± SEM. * *p* < 0.05. DRG: dorsal root ganglion; MrgprA: Mas-related G-protein coupled receptors A; PbTx-1: brevetoxin-1; P-CTX-2: Pacific-ciguatoxin-2; TRPV1: transient receptor potential vanilloid 1.

We assessed the ability of PbTx-1 to sensitize TRPV1 using the agonist capsaicin [69]. PbTx-1 neither significantly modified the percentage of responding cells to capsaicin (85 ± 8.2% compared to control (100%)), nor the amplitude values (116 ± 20% compared to control (100%)) (Figure 3A,B). These results suggest that PbTx-1 does not sensitize TRPV1 in sensory neurons.

We also assessed the ability of PbTx-1 to sensitize MrgprA using the agonist chloroquine [70,71]. The percentage of cells responding to chloroquine was significantly increased (271 ± 53% compared to control (100%)) in the presence of PbTx-1 while the associated amplitude values were not significantly modified (124 ± 30% compared to control (100%)) (Figure 3C,D). These results suggest a sensitization of MrgprA by PbTx-1 in sensory neurons.

**Figure 4:**
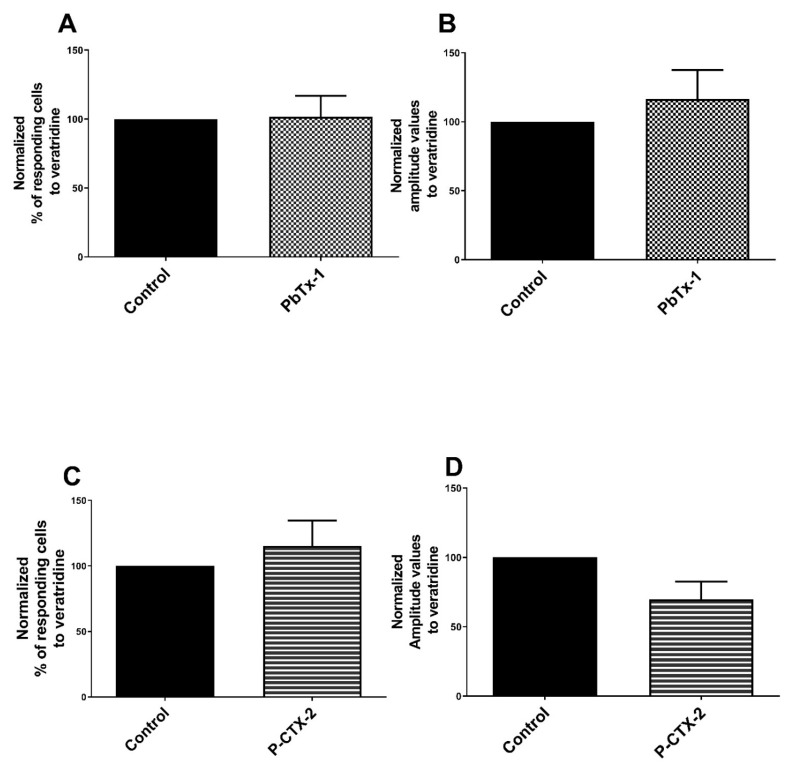
No sensitization of NaV channels by PbTx-1 and P-CTX-2 in DRG neurons. Sensory neurons were pretreated with 1 µM of PbTx-1, 10 nM of P-CTX-2 or vehicles for 20 h. Next, specific agonist of NaV channels, here veratridine 30 µM, was used to assess the sensitization of those channels. Normalized percentages of responding cells (**A**,**C**) and the normalized associated amplitude values (**B**,**D**) of the calcium response induced by veratridine were recorded. Data obtained from 6 (with PbTx-1) and 4 (with P-CTX-2) independent experiments were normalized to vehicle control conditions experiment by experiment. Data were expressed as mean ± SEM and no statistical difference was observed. DRG: dorsal root ganglion; NaV: voltage-gated sodium channel; PbTx-1: brevetoxin-1; P-CTX-2: Pacific-ciguatoxin-2.

We assessed the ability of toxins to sensitize NaV channels using veratridine, which activates these channels without isoform specificity. No significant modification of the two recorded parameters of the calcium response to veratridine was measured after pretreatment with PbTx-1 (102 ± 15% of responding cells compared to control (100%) and 117 ± 21% of the associated amplitude value compared to control (100%)) (Figure 4A,B). No modification of the two calcium parameters in response to veratridine recorded after pretreatment with P-CTX-2 (115 ± 19.4% for the percentage of responding cells; 70 ± 12.7% for the amplitude values compared to control (100%), respectively) (Figure 4C,D). These results suggest that PbTx-1 and P-CTX-2 did not induce overall sensitization of NaV channels, i.e., no sensitization of all NaV channel subtypes taken as a whole.

**Figure 5:**
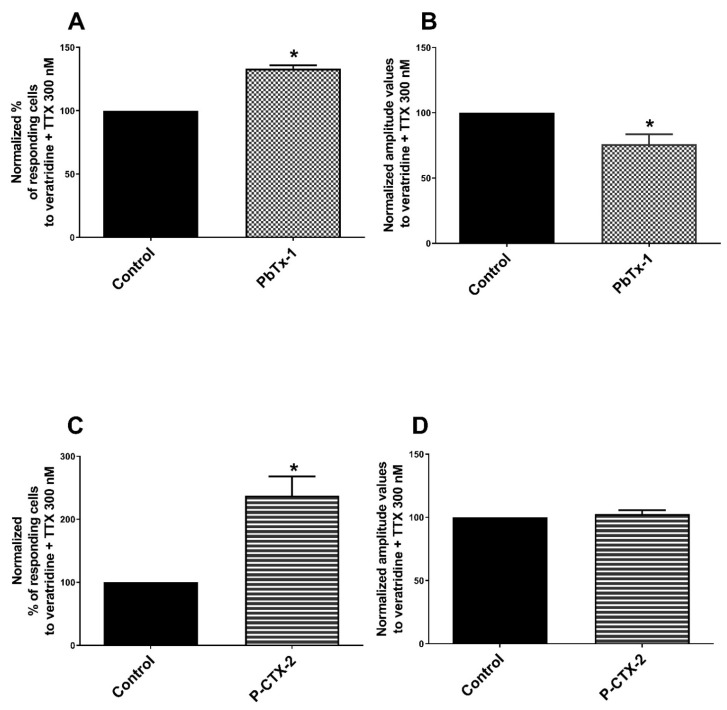
Sensitization of TTX-r NaV channels by PbTx-1 and P-CTX-2 from DRG neurons. Sensory neurons were pretreated with 1 µM of PbTx-1, 10 nM of P-CTX-2 or vehicles for 20 h. TTX 300 nM + veratridine was used to assess the sensitization of those channels. Normalized percentages of responding cells (**A**,**C**) and the normalized associated amplitude values (**B**,**D**) of the calcium signal induced by 300 nM of TTX + veratridine were recorded. Normalized data were obtained from 4 (with PbTx-1) and 3 (with P-CTX-2) independent experiments. Data were expressed as mean ± SEM. * *p* < 0.05. DRG: dorsal root ganglion; PbTx-1: brevetoxin-1; P-CTX-2: Pacific-ciguatoxin-2; TTX: tetrodotoxin; TTX-r NaV: TTX-resistant voltage-gated sodium channel.

In another series of experiments, we assessed the ability of the toxins to sensitize TTX-r NaV channels. TTX was used at 300 nM, which inhibits only TTX-s neuronal NaV channels [47,66] and allows toxin sensitization TTX-r NaV channels to be investigated. The calcium response induced by veratridine in the presence of TTX (pretreatment with 300 nM for 10 min prior to veratridine injection) was assessed. After PbTx-1 pretreatment, the percentage of cells responding to veratridine in the presence of 300 nM TTX (i.e TTX-r NaV channels) was significantly increased (133 ± 2.6% of the control value) and the amplitude value was significantly decreased (76 ± 7.4% of the control) (Figure 5A,B). P-CTX-2 induced also a strong and significant increase in the percentage of cells responding to veratridine + 300 nM of TTX (238 ± 30.5% compared to control condition (100%)) and did not change the amplitude values (103 ± 2.9% compared to control condition (100%)) (Figure 5C,D). These results suggest a sensitization of TTX-r NaV channels by PbTx-1 and P-CTX-2.

### 2.2. Increase in the release of NGF and BDNF in the Supernatant of Homologue Co-Culture Treated by P-CTX-2

**Figure 6:**
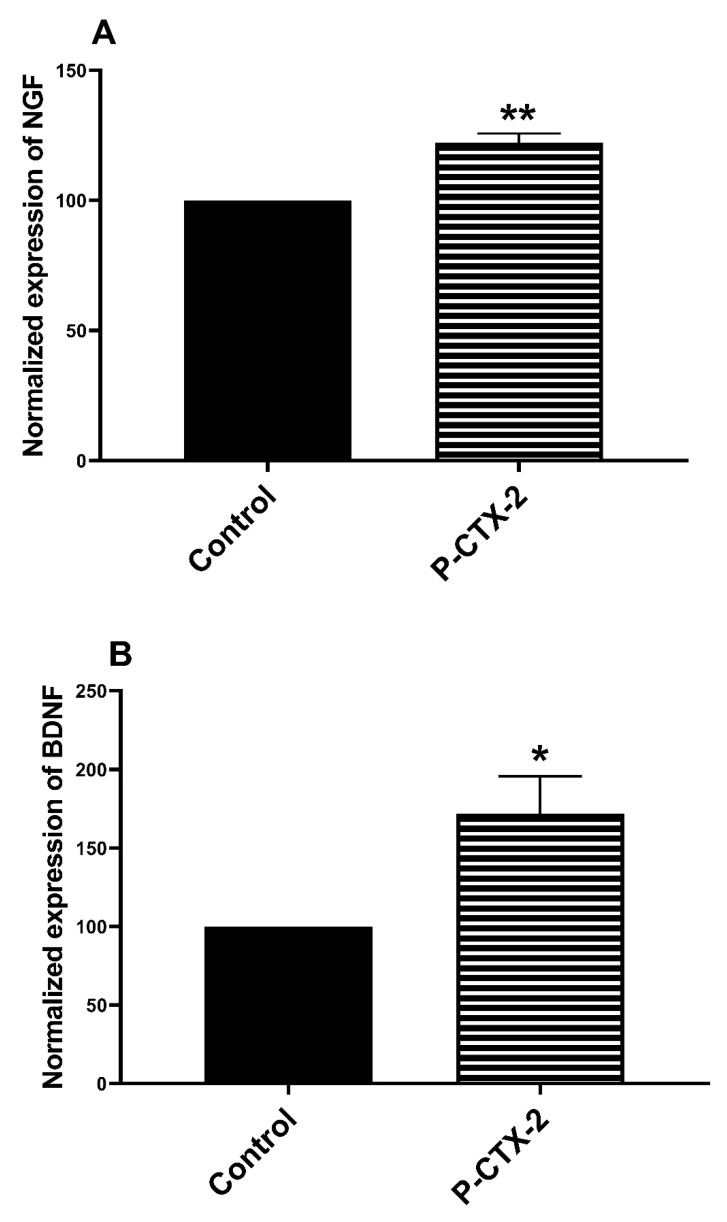
Normalized levels of NGF (**A**) and BDNF (**B**) in the supernatant of homolog co-culture of rat sensory neurons and 10 nM of P-CTX-2. Co-cultured cells were treated with 10 nM of P-CTX-2 or control for 24 h. Data obtained from 4 independent experiments were normalized to vehicle control conditions, expressed as mean ± SEM. * *p* < 0.05 and ** *p* < 0.01. BDNF: brain-derived neurotrophic factor; NGF: nerve growth factor; P-CTX-2: Pacific-ciguatoxin-2.

In the skin, neurotrophins are released by free intra-epidermal nerve endings and also keratinocytes [72,73,74]. To better understand the neuro-epithelial interactions and to identify the soluble mediators potentially involved in P-CTX-2-induced sensitization of receptors, we quantified neurotrophins from our homolog co-culture model of rat sensory neurons and keratinocytes. The release of NGF in the co-culture supernatant was significantly increased to 122 ± 3.5% after treatment with 10 nM of P-CTX-2 compared to control condition (Figure 6A). The release of BDNF was also increased 172 ± 28.9% after pretreatment with 10 nM of P-CTX-2, compared to control condition (Figure 6B).

## 3. Discussion

The present study highlights the ability of PbTx-1 and P-CTX-2 to sensitize several sensory ion channels/receptors in DRG neurons from newborn rats. TRPA1, TRPV4, TTX-r NaV channels, PAR2, MrgprC and MrgprA were sensitized by PbTx-1 and also TRPA1, TTX-r NaV channels, TRPV4 by P-CTX-2. In contrast, TTX-s NaV channels and TRPV1 were not sensitized by PbTx-1 and/or P-CTX-2. NGF and BDNF levels were increased in our homologue co-culture model after treatment with P-CTX-2, representing potential mediators of the sensitization of the receptors/ion channels. This sensitization is one of the mechanisms that may contribute to the sensory disorders induced by both toxins in humans. The results provide new information on the pathophysiology of sensory symptoms in CFP and NSP.

### 3.1. TRPA1 Channel Is Sensitized by PbTx-1 and P-CTX-2 in Rat Sensory Neurons

Our results show that 10 nM of P-CTX-2 and 1 µM of PbTx-1 sensitized TRPA1 to the pharmacological agonist JT010. Our results are consistent with a previous in vivo work showing that P-CTX-1 sensitizes TRPA1 to cold in C-fibers without direct activation [46]. As P-CTX-1-induced cold allodynia involves TRPA1, these results demonstrate a major role of sensitization in sensory disorders induced by CTX. TRPA1 has a major role in pain transduction, notably evoked by PAR2 [54,55] or in nonhistaminergic chronic pruritus by Mrgpr activation [71,75]. However, two studies showed the lack of involvement of TRPA1 in P-CTX-2-induced SP release from an in vitro co-culture model [76] and in P-CTX-1-induced CGRP release from ex vivo mouse skins [29]. Those discrepancies could stem from experimental procedure differences (e.g., absence of pharmacological or cooling stimulus, timing used…) to record CTX-induced sensitization of TRPA1. These data suggest that TRPA1 sensitization by CTX and PbTx is a major component of the sensory disorders occurring during CFP and NSP.

In addition, our results revealed an increased release of NGF in the supernatant of co-cultures treated with P-CTX-2. NGF is able to induce TRPA1 expression in DRG neurons after injury or in an inflammatory context [77,78]. The mechanism includes P38 mitogen-activated protein kinase (MAPK) activation leading to the development and maintenance of cold hyperalgesia [76]. Thus, NGF is a potential mediator of the TRPA1 sensitization induced by P-CTX-2 and PbTx-1.

### 3.2. Sensitization of PAR2 and/MrgprC by PbTx-1 in Rat Sensory Neurons

We demonstrated that PbTx-1 pretreatment induced an increase in the percentage of cells responding to SLIGRL, which was able to activate PAR2 and MrgprC. Further experiments are needed to define the part of each receptor (PAR2 and MrgprC) sensitization by PbTx-1 and P-CTX-2. MrgprC is a member of the Mrgprs nearly exclusively expressed in sensory neurons [77] with important roles in itch [63,78]. PAR2 is mainly localized in small and medium DRG sensory neurons [79], particularly in peptidergic neurons [57] which are the sensory neurons mainly activated by CTX [46]. In previous work, we showed that P-CTX-2 and PbTx-1 activate PAR2 and increase the activity of the PAR2-activating protease cathepsin S in sensory neurons [26,28]. P-CTX-2 and PbTx-1 induced PAR2 internalization 20 min after exposure [26,28]. The calcium response to trypsin resensitized after 60−90 min suggests that PAR2 is re-expressed at the plasmic membrane from preformed pools of receptors after its rapid internalization [80]. The treatment of DRG neurons by PAR2-activating proteases was shown to increase PAR2 expression within a few hours [81]. Taken together, these data suggest that PAR2 sensitization by 20 h treatment of P-CTX-2 and PbTx-1 involves an increased PAR2 expression at the plasma membrane subsequent to its activation.

### 3.3. Sensitization of TRPV4 Channels by PbTx-1 and P-CTX-2 in Rat Sensory Neurons

In our study, we show that TRPV4 is sensitized by PbTx-1 and P-CTX-2. P-CTX-2 significantly increased the amplitude of the response to GSK-1016790A without modification of the percentage of responding cells, while PbTx-1 significantly increased the percentage of responding cells without modification of the response amplitude. Mechanisms could differ between the two toxins. Further experiments are needed to explain these differences between the two toxins.

TPRV4 is expressed by one out of four of rat sensory neurons [82], mainly in Aδ fibers and C fibers [83]. PAR2 activation leads to TRPV4 sensitization [55,84] by both biased and canonical pathways. We showed that P-CTX-2 activates PAR2 [26], PbTx-1 activates PAR2 by both biased and canonical pathways, and PbTx-1 activates both PAR2 and TRPV4 in a common pathway [28]. The latter finding suggests a PAR2-dependent TRPV4 sensitization by PbTx-1. The sensitization of TRPV4 shown in this study by both P-CTX-2 and PbTx-1 is consistent with this hypothesis.

### 3.4. MrgprA Is Sensitized by PbTx-1 in Rat Sensory Neurons

The rat MrgprA (MrgprA3 in mouse) is expressed by a small number of DRG sensory neurons (around 4% of DRG sensory neurons) and is activated by chloroquine [78]. Our results showed that PbTx-1 markedly sensitizes MrgprA in rat sensory neurons. Given the major role of MrgprA in pruritus, our results suggest that sensitization could have a major role in CFP pruritus.

The sensory neurons of rat DRG expressing MrgprA [85] also express a higher density of voltage-gated K^+^ (K_v_) channels than other sensory neurons [85]. K_v_ channels are crucial in the maintenance of the rest state and in the repolarization phase after depolarization [86]. The hyperexcitability of nociceptors by an increase in NaV channel activity and a decrease in K_v_ channel activity is involved in neuropathic pain [87] and is induced by P-CTX-2 and PbTx-1 [21,23]. The neuron subtype expressing MrgprA could play a particular role in the neurotoxin mechanism induced in DRG neurons.

### 3.5. TRPV1 Channels Is Not Sensitized by PbTx-1 in Rat Sensory Neurons

TRPV1 sensitization by PbTx-1 was shown in frog oocytes transfected with TRPV1 [88]. Both NGF [89,90,91] and PAR2 [49,55,62] are well-known for sensitizing TRPV1. However, TRPV1 was not sensitized by PbTx-1 in our model. This result is consistent with previous data showing that TRPV1 is not required for CGRP release in a murine skin model treated by P-CTX-1 [46]. TRPV1 is not involved in P-CTX-2-induced calcium response in sensory neurons or SP release in our co-culture model [74].

### 3.6. TTX-rNaV Channels Are Sensitized by PbTx-1 and P-CTX-2 in Rat Sensory Neurons

PbTx-1 and P-CTX-2 induced a significant increase in the percentage of cells responding to veratridine in the presence of 300 nM TTX, which suggests that both toxins sensitize TTX-r NaV channels by increasing their expression. However, the percentage of responding cells and the amplitude values of the response to veratridine alone were not significantly changed, suggesting that NaV channels as a whole are not sensitized by both toxins. These results highlight the crucial role of TTX-r NaV (especially NaV1.8 and NaV1.9) channels in C and Aδ sensory neurons [92,93], and suggest the role of their sensitization, in the sensory effects induced by those neurotoxins.

Neurokinin 1 receptor (NK1R) activation by SP potentiates NaV1.8 sodium currents in rat DRG neurons by a protein kinase C (PKC)-dependent mechanism probably participating to hyperalgesia [94]. We previously demonstrated that PbTx-1 and P-CTX-2 increase SP release in our co-culture model [26,28]. Together, these data suggest that the SP/NK1R axis could be involved in P-CTX-2-induced sensitization of TTX-r NaV in sensory neurons. NGF is another potential mediator of NaV1.8 sensitization because the NGF level is increased in the supernatant of the co-culture treated with P-CTX-2 in this study. Hyperalgesia induced by NGF in the murine model is mediated by the NaV1.8 [95]. Direct phosphorylation of NaV1.8 by MAPK P38 increases current density at the membrane, contributing to inflammatory and neuropathic pain [96]. The present data suggest that NaV1.8 is a major TTX-r NaV channel sensitized by CTX probably in SP- and/or NGF-dependent mechanisms.

### 3.7. Increase of NGF and BDNF Levels in Co-Culture Model

Neurotrophins, including NGF and BDNF, are key actors of sensitization in sensory neurons [42,43]. Our study shows that P-CTX-2 increased levels of BDNF and NGF in our co-culture model.

BDNF is mainly expressed in small-to-medium sized peptidergic primary sensory neurons (expressing CGRP or SP) [97,98,99]. BDNF level is increased following both inflammation and nerve injury [100,101,102,103,104]. Local treatments with an anti-BDNF antibody reduced pain behavior in several neuropathic pain models [105,106] highlighting the crucial role of BDNF in pain. A transcriptomic study performed in a primary culture of cortical neurons, showed that BDNF gene expression is upregulated after 72 h of cigautoxin-3C treatment [107]. A study in DRG sensory neurons showed that prolonged exposure to BDNF modulates TRP channel expression contributing to chronic pain [108], suggesting a role of BDNF in the sensitization of these sensory channels. Together these data suggest that BDNF could contribute to the sensitization of TRPA1 induced by P-CTX-2 and PbTx-1. However, a recent paper suggested that primary afferent-derived BDNF normally contributes only minimally to the processing of pain and itch [109].

NGF is able to increase NaV1.8 [95], TRPV1 [110,111], TRPA1 [75,76] expression and/or activity in sensory neurons and contribute to hyperalgesia following nerve injury or peripheral inflammation [112]. Increased levels of NGF by P-CTX-2 suggest that NGF is a good candidate for direct ion channel/receptor sensitization induced by those neurotoxins. In addition, 35−40% of adult rat sensory neurons expressed the tropomyosin receptor kinase A (trkA, the specific high-affinity receptor of NGF) [113,114]. TrkA-positive sensory neurons are partially co-expressed with CGRP [115], which are mainly targeted by CTX [46]. NGF increases SP and CGRP levels in skin-innervating sensory fibers [116], BDNF mRNA levels in DRG following peripheral inflammation [117,118] and TrkA mRNA in the sensory neuron-like PC12 cells [119]. Thus, the involvement of NGF in the sensitization of ion channels/receptors could also be an indirect effect via increased BDNF, SP and CGRP levels.

In this study, we identified the ability of PbTx-1 and P-CTX-2 to sensitize several sensory receptors in rat DRG neurons. The present data is subject to several limitations. The intervariability of the primary sensory neuron cultures may explain the variability observed in the responses to the different analog neurotoxins. Increased numbers of experiments could decrease the interexperimental variability but due to the limited availability of P-CTX-2, the repetition of the experiments was limited. It should be noted that in vitro results obtained in rodent DRG neurons may be considered with caution because they do not integrate the complete environment, including the spatial and functional organization of DRG neurons and fibers, immune and epithelial cells, and differences might exist between rodents in vivo and humans [120,121].

## 4. Materials and Methods

### 4.1. Reagents

PbTx-1 and TTX were purchased from Latoxan (Valence, France). One µM of PbTx-1 was considered as a relevant concentration, given its relative potency compared with P-CTX-1 and P-CTX-2 [21,122] and our studies [26,28]. Pure P-CTX-2 was isolated from moray eel (*Gymnothorax javanicus*) livers as previously described [122]. A 1.15 mM stock solution of PbTx-1 was prepared in pure methanol (MeOH), while a 10 µM stock solution of P-CTX-2 was prepared in MeOH: water (1:1). Aliquots of PbTx-1 stock solution were next dried and resolubilized in culture medium. Culture media were supplemented with 100 µg/mL of Normocin (InvivoGen, Toulouse, France). Keratinocyte-serum free medium (KSFM) with L-glutamine, epidermal growth factor, and bovine pituitary extract provided by Life Technologies (Saint Aubin, France) to obtain a so-called complete KSFM. Dulbecco’s Modified Eagle Medium (DMEM) and DMEM plus Ham’s F12 media (DMEM:F12) were purchased from Lonza Group Ltd. (Basel, Switzerland), veratridine, JT010, capsaicin, SLIGRL, and chloroquine were purchased from Sigma-Aldrich (Saint Quentin Fallavier, France) while GSK-1016790A was provided by Abcam (Cambridge, United Kingdom). To study TTX-r NaV sensitization, neurons were pretreated with TTX 300 nM, which inhibits only TTX-s NaV channels [27,47,93], before treatment with the agonist veratridine. Agonist targets and concentrations used are summarized below in the Table 1.

### 4.2. Cell Culture

Animal experiments were approved by local authorities and in accordance with the French Ministry of Agriculture and the European Communities Council Directive 2010/63/UE. They were approved by the veterinary services of the Departmental Directorate for the Protection of Populations of Finistere and the Animal Welfare Structure of the University of Brest, France.

Rat sensory neurons were obtained from DRG of neonatal rats as previously described [27]. Briefly, DRG were extracted from newborn Wistar rats between 2 and 5 days after birth, then enzymatically (collagenase IV, 200 units/mL) and mechanically dissociated. DRG suspension was filtered through 70 µm cell strainer before seeding in 96-well plates or on glass coverslips coated with poly-l-lysine (PLL).

Rat keratinocytes were obtained from neonatal rat skin as previously described [127] with minor modification including the dispase (20 UI/mL) used for enzymatic digestion overnight at 4 °C. Keratinocytes between 1 and 3 passages were maintained in complete KSFM for 24 h and then, differentiated for 14−16 h in mixture of DMEM/DMEM:F12 (1:1).

The co-culture model was performed as previously described [27] with minor modifications. Briefly, DRG neurons from one newborn rat were seeded in 10 wells of 96-well plates in DMEM/DMEM:F12 (1:1) supplemented with Normocin (100 µg/mL), B27 (20 µL/mL), NGF (100 ng/mL), insulin (4 µg/mL), BDNF (20 ng/mL) and hydrocortisone (10 ng/mL). After 3−5 days of culture, the medium was removed, and 20,000 rat non-differentiated keratinocytes per well were seeded in complete KSFM. Co-cultures were maintained for 24 h at 37 °C in a 5% CO_2_ humidified atmosphere to allow keratinocyte attachment and then the medium was replaced by DMEM/DMEM:F12 (1:1) to induce keratinocyte differentiation for 14−16 h.

### 4.3. Single-Cell Calcium Video Imaging

DRG neurons were cultured on glass coverslips coated with PLL and maintained at 37 °C for 24 h. Then, DRG neurons were treated for 20 h either with 1 µM of PbTx-1, 10 nM of P-CTX-2 or vehicle controls. Later, DRG neurons were placed in a recording buffer (135 mM NaCl, 5 mM KCl, 1 mM MgCl_2_, 1.8 mM CaCl_2_, 10 mM HEPES, 10 mM glucose and pH adjusted at 7.45 with NaOH) and loaded with 4 µM of Fura-2/AM (Molecular Probes, Invitrogen, Cergy Pontoise, France) for 30 min at 37 °C. After washing, ratiometric images of calcium signals (340/380 nm) were obtained with the microscope IX71 Olympus equipped with a monochromator illumination system (Polychrome V, TILL Photonics). Emission at 510 nm was captured through a 415 DCLP dichroic mirror, by a 14-bit CCD camera (EXiBlue, Qimaging). Image acquisition and analysis were performed with the Metafluor 6.3 software (Universal Imaging, West Chester, PA, USA) at room temperature.

At least 50 to 150 regions of interest were defined with single neuron for each condition of each experiment. Agonist was injected manually and data were recorded for several minutes. Background subtraction and calculation of the resulting 340/380 ratio images were performed offline. Calcium entry was quantified after value normalization (with the formula ΔF/F0 = (F−F0)/F0, where F is the ratiometric value at a given moment and F0 is the baseline (average of values before agonist injection)). Cells were considered to respond to the agonist when they exhibited a ΔF/F0 increase of at least 0.15. To study the effect of pretreatment with P-CTX-2 or PbTx-1 on the response to the agonist, two parameters were recorded: the percentage of responding cells and the maximum amplitude value associated. The sensitization was assessed by measuring the ability of pretreatment with PbTx-1 or P-CTX-2 to increase percentages of cell responding to the specific agonist and/or the associated amplitude values, compared with vehicle pretreatment. We used normalized data, due to the interexperimental variability in the responses of primary neurons, considering as 100% the average data obtained with control condition for each experiment. The “n” represents the number of independent experiments in which 50 to 150 neuronal cells have been integrated to the calcium analysis. Data were expressed as mean ± standard error of the mean (SEM) of at least 3 separate experiments.

### 4.4. Magnetic Bead-Based NGF and BDNF Immunoassays

Co-cultured sensory neurons and keratinocytes were maintained between 5 to 7 days, washed twice with medium without supplement and then treated for 24 h with 10 nM of P-CTX-2 or equivalent of MeOH (vehicle control). Supernatants of the co-culture were collected and stored at -80 °C. Neurotrophin levels were measured in the supernatants using the magnetic bead-based assay (MAGPIX—Luminex, Austin, TX, USA) and the mono-analysis kits for BDNF (RMYOMAG-88K-01, Merck-Millipore, Burlington, VT, USA) and NGF (HAD2MAG-61K-01, Merck-Millipore, Burlington, VT, USA) according to the manufacturer’s instructions. Data were collected using MAGPIX instrument (Luminex, Austin, TX, USA) and xPONENT Software Version 4.3 (Luminex, Austin, TX, USA).

### 4.5. Data Analysis

The statistical analyses were conducted with GraphPad Prism 6.0 (San Diego, CA, USA). The details are provided in the figure legends. Shapiro−Wilk normality test was performed and in the case of non-normal distribution, a non-parametric approach was chosen. Statistical analysis has been performed using a t-test. The differences were considered statistically significant with *p* < 0.05.

## 5. Conclusions

This study shows that the marine neurotoxins P-CTX-2 et PbTx-1 are able to sensitize in vitro several receptor/ion channels in the DRG neurons from newborn rats, including TRPA1, MrgprA, MrgprC/PAR2, TRPV4 and TTX-r NaV channels. In contrast, TRPV1 and TTX-s NaV channels are not sensitized by PbTx-1 and P-CTX-2. Similar to the sensory neuropathies of other origins, the sensitization of those receptor/ion channels may contribute to the development and maintenance of sensory symptoms occurring during CFP and NSP. An increased level of NGF and BDNF after P-CTX-2 treatment in our homologue co-culture model suggests they are potential mediators of this sensitization. Our results provide new knowledge on the pathophysiology and could be very useful for the development of therapeutic options for CFP and NSP.

## Figures and Tables

**Table 1:** Target receptors/ion channels and their corresponding agonists: Concentrations used for sensitization study.

Target(s)	Agonist/Antagonist	Concentration Used	References
NaV	Veratridine	30 µM	[12,123]
TTX-r NaV	Veratridine/TTX	30 µM/300 nM	[47,93,123]
PAR2/MrgprC	SLIGRL	100 µM	[66,67]
TRPA1	JT010	10 µM	[65,124]
TRPV1	Capsaicin	200 nM	[125,126]
TRPV4	GSK-1016790A	10 nM	[68]
MrgprA	Chloroquine	2 mM	[78]

## Data Availability

The data presented in this study are available on request from the corresponding author.
